# Do circular economy, public-private Partnership and carbon policy manage the environmental stress? Developed countries' situation under the Prism of COP27

**DOI:** 10.1016/j.heliyon.2024.e33532

**Published:** 2024-06-24

**Authors:** Daniel Balsalobre-Lorente, Syed Ale Raza Shah, Rena Huseynova

**Affiliations:** a Department of Applied Economics, University of Castilla La Mancha, Spain; b Department of Management and Marketing, Czech University of Life Sciences Prague Faculty of Economics and Management, Prague, Czech Republic; c UNEC Research Methods Application Center, Azerbaijan State University of Economics (UNEC), Istiqlaliyyat Str. 6, Baku 1001, Azerbaijan; d Western Caspian University, Economic Research Center (WCERC), Baku, Azerbaijan; e School of Economics & Finance, Xi'an Jiaotong University, Xian, 710061, China; f Department of Digital technologies and applied informatics, Azerbaijan State University of Economics (UNEC), Istiqlaliyyat Str. 6, Baku 1001, Azerbaijan

**Keywords:** *Policy stability*, *Climate change*, *Circular economy*, *Public-private partnership*, *COP27*, *Developed nations*

## Abstract

Since the Industrial Revolution, the economies have played well to make progress in economic growth. Besides, rapid growth has brought severe challenges, and environmental degradation is one of them. Therefore, the globe has introduced several green initiatives, such as the Kyoto Protocol, the Paris Agreement, and the Sustainable Development Goals, but the problem remains intact. Specifically, this study focuses on ***COP27*** and highlights the key challenges and their best solutions. Undoubtedly, most nations have tried to meet their settled targets by 2030, but these have different priorities to facilitate their populace. Therefore, international cooperation has been introduced as a logical solution to collaborate across borders or within the region to deal with sustainability themes. However, developed nations have environmental problems due to industrial, income, and population growth, directly associated with environmental risks. Thus, under the SDGs, this empirical research tries to cover the critical problems (income, population aging, & industrial development) and their best alternative (public-private partnership, emission taxes & circular economy) to minimize environmental issues. Similarly, the current study utilizes an advanced series of estimators to investigate the study's objective for 17 developed nations from 2000 to 2021. Investigated outcomes describe income, population aging, and industrial activities that bring carbon emissions. Conversely, carbon policy and public-private partnerships support the sustainability theme for specified economies. Under the base model, the circular economy declines the environmental pressure by 0.016 %, 0.002 %, and 0.019 %, respectively, under the specified estimators. Moreover, this empirical research investigates the mediating role of carbon policy, public-private partnership & circular economy on industrial development. It brings a significant decline in emissions only for carbon policy & circular economy. However, this study also proposes some green policies to become clean & green shortly.

## Highlights

• Key environmental determinants have been proposed under the COP27 theme.
• Socio-economic and development factors contribute to environmental stress.
• The circular economy and carbon policy are the best sustainability solutions.
• The PP's mediating role has an insignificant impact on environmental sustainability.
• Some green policies have been suggested to overcome environmental pollution.

## Abbreviation

ED: Environmental degradation
CO_2_: Carbon emission
PP: Public-private partnership,
ECI: Economic complexity index
PA: Population aging
ID: Industrial development
CE: Circular economy
CP: Carbon policy
SDGs: Sustainable Development Goals
CSD: Cross-Sectional dependence
CADF: the covariate-augmented. Dickey-Fuller
CIPS: Cross-sectionally augmented Im-Pesaran-Shin
AMG: Augmented Mean Group,
CCE-MG: Common Correlated Effect Mean Group,
CS-ARDL: Cross-Sectional Autoregressive Distributive Lag model

## 1 1- Introduction

Over the last few decades, lifestyles have dramatically changed and caused environmental stress [1]. For rapid development, economies have made significant efforts to make industrial transition from small-scale production units to well-organized ones. However, the fast progress and its sweet outcomes on the overall economy have compelled us to voluntarily ignore negative consequences on humankind [2]. Thus, for the first time, Carson has tried to awaked the nations from green outcomes of rapid growth and introduce the environmental issues [3]. Emerging, developing, and developed economies are trapped in environmental stress and sorting out some of the best alternatives [4]. For instance, the globe has introduced numerous essential suggestions to combat rising emissions, such as the Paris Agreement, the Kyoto Protocol, and sustainable development goals (SDGs), but the problem remains intact [5]. Thus, the UN has tried to call every country on one page and is compelled to fight for environmental sustainability at minimum development loss. Recently, COP27 has been organized to highlight the importance of a sustainable future. Therefore, the UN has focused on four different themes: environmental mitigation, green methods adoption, green financing, and collaboration, which may help to secure the present & future generations from environmental harm. Besides, the general arguments regarding environmental concerns and their influences on social life compel analysts to clear the fundamental problems that must be addressed in forthcoming decades. Therefore, this study tries to introduce the key problems that are leading cause of environmental stress across the different regions.

Rapid economic progress has remained a core factor in the leading environmental problems [6]. Since 1970, nations have made significant progress in boosting income per capita at the cost of environmental pollution. Undoubtedly, economies have attained rapid economic progress, but they offset the environmental harms. For the first time, this suspense has been broken by Ref. [7] by investigating the critical connection of emissions with development. In the summary, they validated the inverted U-shaped EKC-hypothesis for specified variables. However, this theme refers to a significant increase in income bringing massive environmental pollution, but after its threshold level, it caused a decline [[8], [9], [10], [11]]. Secondly, the current study focuses on the social factors that continuously degrading environmental quality. In this era of development, human activities are increasing environmental pollution. Currently, the globe is facing mainly environmental issues as per population structure. Interestingly, with rapid economic growth, economies have paid particular attention to the quality of life that may increase social well-being. Thus, in close years, the influence of population growth and its severe consequences on sustainability have been studied well. Although the different measures of population structure, such as population growth, urbanization, and male & female population, have been debated well, the most influential population measure is voluntarily ignored. Therefore, it is necessary to focus on population aging, which could be a leading factor influencing sustainability. Thirdly, this study focuses on industrial development, which has remained a critical global challenge due to its severe environmental effects. Economies have tried to make significant progress in the industrial sector for rapid improvement. The industrial sector across the nations is in a transition phase that faces numerous challenges, such as environmental standards, green production, efficient labor, and cheap raw materials. However, it is a common belief that most economies rely on traditional energies in their production activities due to conventional set-ups. Thus, the demand side urges the utilization of energy-intensive inputs in production activities that may cause long-term environmental harm. Besides the air pollution, the industrial sector's water, soil, and noise pollution cannot be ignored from this race.

However, under such stresses, the leading economies have decided to fight for sustainability and proposed ***several green initiatives***. In these green initiatives, environmental regulations have received immense attention. Interestingly, a hasty rise in energy utilization and its consequences on environmental quality compel policy analysts to introduce some environmental taxes, and the carbon tax being considered is one of them [12]. However, the carbon tax has two aspects that can raise environmental sustainability [13]. Firstly, it increases energy prices, raising the enterprises' costs and significantly declining emission-intensive productive activities. Furthermore, the carbon tax is uncertain regarding consumption patterns because enterprises never pay external costs and try to shift them to consumers through higher prices. Thus, this method is considered to earn fiscal revenue and control emissions. Therefore, in the era of development, a carbon tax could be the best solution to resolve the emissions problems at the domestic level. More interestingly, in recent years, policy analysts have tried to pay magnificent attention to public-private partnerships under the SDGs theme. Therefore, the Kyoto Protocol standards have voluntarily compelled economies to shift their energy preferences from traditional to modern. Resources available for energy transition have not contributed immensely; thus, higher authorities are trying their best to promote public-private partnerships in eco-friendly projects. Also, the existing system has ignored its importance and the effects of green spillover on the overall economy. Thus, under the theme of the UN development plans, the public-private partnership has been considered a last resort to deal with sustainable development at lower environmental costs. Therefore, under the public-private partnership, the economy sectors cannot be ignored as the best solution for environmental sustainability. Finally, this study considers the circular (CE) economy as the best alternative to deal with rising environmental stress. Due to rational behavior, CE has become a prominent solution for environmental change, biodiversity loss, and wastage. Similarly, the European Union reports that more than half of environmental pollution comes from production activities. Consequently, CE can be considered the best alternative that deals systematically with production & consumption activities; therefore, it urges to reutilize available resources efficiently and return them to the economy. This method secures the environmental quality and helps reduce companies' costs. Due to such supportive behavior, most developed economies are moving towards the circular economy. This study focuses on these three uninterrupted solutions to deal with environmental deterioration.

However, by having a detailed logical debate regarding the fundamental problems and their proposed best solution, the present research focuses on the ***top circular economies*** from 2000 to 2021. However, the present study contributes to the existing literature in the following ways. **Firstly,** the present research utilizes the economic complexity index (ECI) to measure development activities comprehensively. In earlier studies, most of the time, series and panel data studies have used income as the best development activity measures, but they cannot deliver the actual influence of development plans and their harmful impact on a sustainability level. Therefore, the present study utilizes the ECI to overcome the existing ambiguity among practitioners and suggest green measures for future perspectives. Secondly, it is a common belief that a social factor such as population growth is also a key determinant of environmental quality. Due to daily human & economic activities, the massive cost of energy utilization may cause environmental pollution. Therefore, this study differs from the existing literature because it considers the population aging (15–64 years) to be a core contributor to economic activities. Undoubtedly, other measures, such as overall population growth and old age people, also have a significant role at the domestic level, but this proxy could provide an accurate outcome regarding sustainability. **Thirdly**, the industrial sector has become a priority for developed, emerging, and developing economies to progress significantly. However, it cannot be ignored that this sector is in the transition phase and still needs improvement to become eco-friendly. Due to its incompatibility with green initiatives, it may rely on energy-intensive technologies that would cause energy deficits and raise emissions. **Fourthly,** carbon taxes could be the best alternative to deal with environmental deterioration; therefore, this empirical research tries to add an innovative determinant to the development-environment model. Nowadays, the globe is facing several issues, such as energy price fluctuations, environmental damages, and energy security, and most nations are trying to impose carbon taxes that may work for sustainability. Although carbon taxes positively and negatively impact social well-being, managing them is also critical. Thus, the present research tries to investigate the mitigation role of the carbon tax on top circular economies, and these economies could be leading examples of carbon taxpayers.

**Fifthly,** the crucial role of the public-private sector (PP) in overall sectors is minimal. Therefore, a single effort by one actor would not reduce environmental stress sufficiently [14]. The critical contribution of socio-economic & energy factors to environmental sustainability has been well debated, but the importance of public-private partnerships is missing. This empirical research tries to incorporate this factor as another eco-friendly solution that may produce robust outcomes. **Sixthly,** the circular economy is also an innovative determinant for the environment model; thus, this study believes that re-utilization of wastage secures energy sources and contributes to less environmental harm. Due to its impressive role, policy analysts can utilize it as a green tool to reduce environmental pressure at the domestic level. Finally, this empirical research investigates the mediating role of carbon policy, PP, and circular economy on the industrial sector and examines their response to sustainability. Surprisingly, the mentioned green factors can manage environmental deterioration via the industrial sector and open a new door to involvement in different sectors of the economy. More interestingly, this study utilizes the imperative series of econometric estimators that can deal with all panel data problems. Therefore, this study uses CSD, homogeneity slope, integration, and long-run cointegration tests to validate panel data properties. Furthermore, for the long-run association among the selected variables, this study tries to use the Augmented Mean Group (AMG), Common Correlated Effect Mean Group (CCE-MG), Cross-Sectional Autoregressive Distributive Lag model (CS-ARDL), and Panel Quantile regression.

The remaining sections are classified as Section 2: Literature Review, section 3: Data Collection and Methods, section 4: results & discussion, and section 5: Conclusion and policy recommendations.

## 2 2- Literature Review

The current section is vital to overlook regarding the past studies and their profound comments on core variables. For illustration, this study provides three sub-sections to understand the clear theme regarding carbon policy, public-private partnership, and circular economy with CO2 emission levels. This study provides a detailed summary in the following ways for deep understanding.

### 2.1 Carbon Policy and Environmental Stress

Similarly, the carbon tax policy is directly derived from the Pigovian tax, which describes environmental costs by adding them to total expenses. Therefore, the carbon policy has focused on consumption-based emissions that are emitted from fuel burning, and this policy tries to reduce them efficiently. Thus, under the comparison between carbon & energy policy, carbon policy has remained a catchy phenomenon that secures environmental quality and tries to transition energy from traditional to modern [15]. Whalley and Wigle [16] demonstrated the crucial role of carbon policy in emissions levels and tried to compare it with other taxes; consequently, outcomes described a magnificent role in emissions reduction by carbon policy. Symons et al. [17], investigated the long-term association between carbon policy and carbon pollution in Britain's economy and found a significant role of carbon policy on fossil fuel prices, indirectly increasing overall product prices and hurting purchasing power. Thus, a substantial decline in consumption patterns brings less environmental pollution. Aasness et al. [18], employed the generalized method approach and tried to evaluate the carbon policy's connection with environmental quality in Norway's economy. However, they suggested that a significant increase in carbon policy would efficiently reduce CO_2_ emissions in the long run. Later, Nakata and Lamont [19] tried to work on a comparative analysis whether carbon or energy tax has a more significant role in climate change. Under their suggestion, they found carbon & energy taxes are both feasible to fight for sustainability, but carbon tax plays well to shift energy consumption patterns from coal to natural gas. Floros and Vlachou [20] highly focused on carbon policy and its green spillover effects on environmental quality in Greece's economy. However, they directly linked this theme with the manufacturing industry and found interesting outcomes regarding carbon policy. A significant intervention of carbon policy compelled energy transition from natural gas to renewable energy consumption. Wissema and Dellink [21] evaluated the case study of the Irish economy and tried to check out the long-run connection between carbon energy policy and carbon-intensive energy. Concluding suggestions described that carbon policy significantly reduces the consumption level of coal, a leading cause of environmental stress, and promotes the utilization of green energy in economic activities. Also, Lu et al. [22], demonstrated the key contribution of carbon policy in emission reduction for China's economy and they found a significant improvement in environmental sustainability at minimum growth loss.

**Fig. 1:**
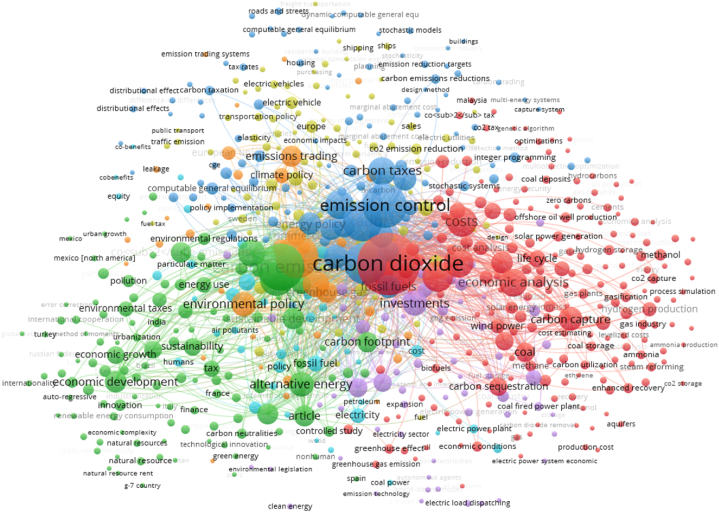
Carbon policy and environmental stress.

Besides the earlier decades and authors' contribution to carbon policy & sustainability level, it is necessary to overview the current opinions of policy analysts regarding carbon policy & environmental pollution. This theme has been debated in recent years, and fresh studies are summarized as follows. Alper [23] investigated the long-term connection of carbon policy with rising emissions levels in 18 selected European economies from 1995 to 2015. He employed the Feasible Generalized least square and showed an inverse association of carbon policy towards the emissions level. However, he has not tried to explain the core logic behind the scene. A case study of EU members' economies Aydin and Esen [24] also attempted to investigate the role of carbon policy on sustainability over the period of 1995–2013. However, they had tried to focus on different taxes, such as taxes on transport, pollution, and resources. They employed the dynamic panel threshold model and found an insignificant connection between environmental tax and emissions level. However, they demonstrated different logic; firstly, the rapid progress in urbanization & growth causes energy-intensive activities and causes fewer roles by carbon policy at the domestic level. However, these activities have been observed chiefly in congested urban areas where environmental regulations remain insignificant. Likewise, a study related to OECD economies Bashir et al. [25], demonstrated the key connection between carbon policy and sustainability level. They utilized the system GMM & panel quantile regression to investigate the core study's objectives. Outcomes described the negative role of carbon policy on rising emissions. However, this association has been explained on behalf of technological innovation and its spillover effect on production activities that bring massive environmental reduction. Khan et al. [26], described the impact of carbon policy on environmental quality in 19 EU economies covering the period of 1995–2019. They employed the most robust estimators and found the emission reduction by carbon policy at lower quantiles. This connection explains that carbon policy has inefficient behavior in emissions reduction. Thus, production units have ignored the environmental regulations; as a result, environmental policy has become insignificant. Cheng et al. [27], demonstrated the long-term impact of carbon policy on the sustainability level of Sweden's economy from 1990 to 2019. They employed the quantile-on-quantile regression and found energy innovation & carbon policy are the most eco-friendly indicators in specified economies. However, carbon policy highly influenced the median quantile and supported the sustainability theme. Telatar and Birinci [28], focused on Turkey's case study over the period of 1994–2019 and tried to investigate the impact of carbon policy on CO_2_ emissions. They utilized the Smooth Transition Autoregressive regression and Markov Switching estimator to evaluate key study themes and found the negative effect on carbon emissions. However, the authors have not sufficiently described the fundamental logic behind the mentioned connection. However, Kirikkaleli [29], a study related to the Canadian economy, also described the role of carbon policy in sustainability levels from 1990 to 2020. However, the outcomes investigated by a non-ARDL estimator showed a significant carbon reduction for the Canadian region. Due to massive environmental taxes, firms have made considerable efforts to cleanse production and bring environmental sustainability to the domestic level. However, the present study also provides the overall studies from 2018 to 2024 regarding the carbon policy and carbon emissions. In the relevant literature, there has seen 888 case studies for the relevant theme. Therefore, the present study provides the co-occurrence bibliometric graph to interlink carbon policies studies to CO_2_ emissions (see Fig. 1).

### 2.2 Public-Private Partnership and Environmental Stress

In recent years, the public-private partnership (PP) has received particular focus from policy analysts in dealing with energy thirst and environmental sustainability. Therefore, it would be surprising if the present study tries to compile its theoretical literature for more clear understanding. Over the last three decades, stakeholders have tried to prove that partnership is the best strategy for sustainable development. For sustainability's sake, the UN has arranged the first meeting in South Africa in 2002. The leading aim of authorities was to ensure partnership across all regions under efficient institutions. Later on, this concept was nominated by the win-win partnership because countries paid magnificent attention to the public-private partnership [30]. At that time, the leading role of partnership was observed to control poverty. Similarly, under the sustainable development theme, the UNDP program has maintained a strong opinion regarding the partnership between energy and water resources. Also, theoretical literature has defined the advantages of PP that would cause sustainable development in the long run. These advantages are access to crucial resources, lower cost, perfect information, rational behavior, efficient utilization of resources, negotiation with experts, and fewer conflicts among the economic actors. Thus, with a short overview of PP's theoretical background, this study provides a detailed empirical review of public-private partnerships and environmental stress.

**Fig. 2:**
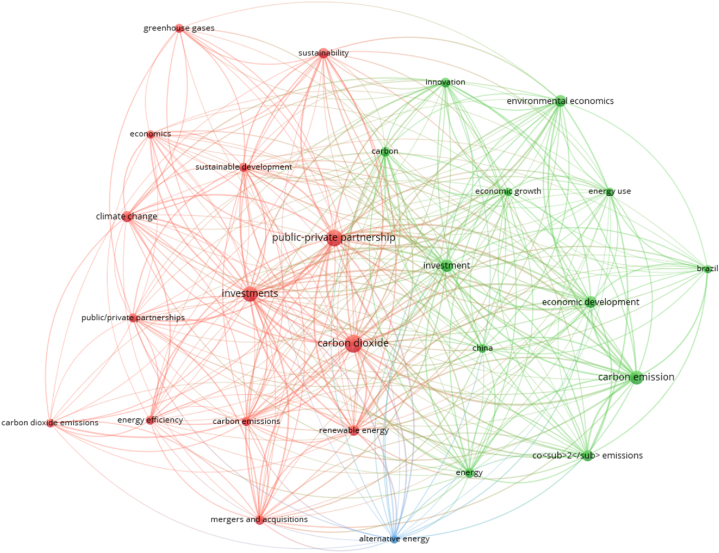
Public-private Partnership and environmental stress.

In the empirical literature, few studies have directly evaluated the long-term impact of public-private partnerships (PP) on environmental sustainability. For instance, Buso and Stenger [31], tried to offer an overview of the PP and its response to climate change. The general arguments provided two points for the existing literature; firstly, PP has seen more preferable initiatives than public product subsidies. Moreover, they argued that PP could be the best strategy if their allocation is based on a fair process over time. Similarly, Khan et al. [32], investigated the impact of PP in the energy sector on environmental pollution in the case of China's economy over the period of 1990–2017. By considering the quarterly data along with GLS, OLS, DOLS, and CCR cointegration tests, they found a rise in environmental stress due to a significant surge in PP. However, they claimed this was observed due to massive investment in non-green energy consumption via PP. Kirikkaleli and Adebayo [33] investigated the core contribution of PP in energy towards the environmental stress in India's economy, covering the time from 1990 to 2015. Using the FMOLS and DOLS, they found a significant contribution to carbon emissions in the long run. They also demonstrated that as per the economic status of the Indian economy, most public-private projects have been organized on emissions-intensive; therefore, the energy sector remained at the traditional pattern. Adebayo et al. [34], they investigated the role of PP in energy and its role in emissions levels for the Asia & Pacific economies over the period of 1992–2015. They utilized the DOLS, FMOLS, and ARDL estimators and found that the significant rise in PP in the energy sector increases emissions. However, they also claimed that at this time, PP had been made to promote overall energy consumption, whether green or non-green energy. Another case study by China Cheng et al. [35], evaluated the role of PP in energy in environmental sustainability by considering 1991–2017. However, the investigated outcomes describe a positive trend in emissions levels due to the rise in PP in the energy sector. However, as summarized earlier, they have not deviated from the prior arguments. Akinsola et al. [36], examined the role of PP in the Brazilian economy's sustainability level via the energy sector. They utilized the DOLS & ARDL estimators to find PP' positive connection with environmental degradation for the specified economy. A case study of Pakistan's economy Zhang et al. [37], also demonstrated the long-run impact of PPPs on ecological deterioration over the period of 1980–2019. They found a positive connection between PP and emissions levels. However, all case studies are summarized based on time series. It is the leading drawback of the existing literature. However, the present study also provides the overall studies from 2018 to 2024 regarding the Public Private Partnership and carbon emissions. The relevant literature has seen 38 case studies for the concerned theme. Therefore, it is necessary to provide the co-occurrence bibliometric graph to interlink PPP to emissions studies (see Fig. 2).

### 2.3 Circular Economy and Environmental Stress

**Fig. 3:**
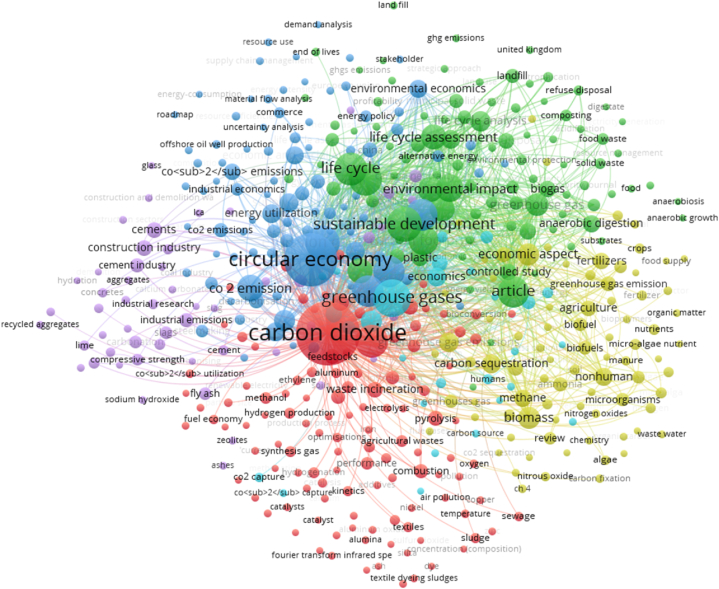
Circular economy and environmental stress.

In recent years, the circular economy has become a prominent source of keeping the environment clean & green. This sub-section summarizes the overall arguments of policy analysts regarding the circular economy (CE) and its spillover effects towards the sustainability level. However, CE has been considered a green instrument to manage waste utilization in three ways, i.e., minimizing wastage, reutilizing, and protecting the ecosystem. Over the last few decades, CE has explained how economies can efficiently utilize their wastages to combat emissions. From the theoretical perspective, American economist Kenneth Boulding first introduced the concept of circular economy in his Spaceship theory [38]. The CE is now considered a new phenomenon behind green development [39]. Thus, CE could be the best solution to reduce over-dependency on non-green energy, which further causes an increase in economic, environmental, and social benefits [40]. However, minimal empirical studies have tried to investigate the connection of emissions with circular economy (CE) across the different regions. Similarly, a time series study for the Angola economy Maria et al. [41], described the interconnection of CE with emissions level. However, they adopted the landfill method and found the direct contribution of CE to biodiversity losses. Razzaq et al. [42], demonstrated the key contribution of CE on the sustainability level in the United States economy covering the period of 1990–2018. They utilized the ARDL estimator for the study's objectives and found a significant decline in environmental stress via waste re-utilization. This connection was found due to the substantial penetration of the recycling method rather than the land-filing method in the circular economy that brought sustainability at the domestic level. In late numerous case studies have investigated the contribution of circular economy in different aspects such as energy and economic but not specified to sustainability theme [please see: study of Nigeria Olujobi et al., [43]; study of Bangladesh Roy et al., [44]; study of Malaysia Chew et al., [45]; study of China Ma et al., [46]; and study of Fly Ash Hou et al., [47]. Similarly, the present study also provides the overall studies from 2018 to 2024 regarding the Public Private Partnership and carbon emissions. The relevant literature has seen 478 case studies for the concerned theme. Therefore, it is necessary to provide the co-occurrence bibliometric graph to interlink circular economy to emissions studies (see Fig. 3).

Similarly, Table 1 describes a detailed summary of past studies directly connected with mentioned literature classifications.

**Table 1:** Literature summary.

Authors	Region	Technique	Outcomes
Carbon Policy and Environmental stress			
Alper, (2017)	18 selected European economies	FGLS	CP ↓ CO_2_
Aydin and Esen, (2018)	EU members' economies	DPTM	CP ↓ CO_2_
Bashir et al. (2020)	OECD	System GMM & PQR	CP ↓ CO_2_
Khan et al. (2021)	19 EU economies	PQR	CP ≠ CO_2_
Cheng et al. (2021)	Sweden	QQR	CP ↓ CO_2_
Telatar and Birinci (2022)	Turkey	STAR, MSV	CP ↓ CO_2_
Kirikkaleli, (2023)	Canadian economy	non-ARDL	CP ↓ CO_2_
Public-Private Partnership and Environmental Stress			
Buso and Stenger, (2018)	Review Study	N/A	PP ↓ CO_2_
Khan et al. (2020)	China	GLS, OLS, DOLS, and CCR	PP ↑ CO_2_
Kirikkaleli and Adebayo, (2021)	India	FMOLS & DOLS	PP ↑ CO_2_
Tomiwa Set al., (2021)	Asia & Pacific economies	DOLS, FMOLS, and ARDL	PP ↑ CO_2_
Cheng et al. (2021)			PP ↑ CO_2_
Akinsola et al. (2022)	Brazil	DOLS & ARDL	PP ↑ CO_2_
Zhang et al. (2023)	Pakistan		PP ↑ CO_2_
Circular Economy and Environmental Stress			
Maria et al. (2020)	Angola	Land fill method	CE ↑ CO_2_
Razzaq et al. (2021)	The USA	ARDL	CE ↓ CO_2_
Olujobi et al. (2022)	Nigeria	ARDL	CE ↑ CO_2_
Roy et al. (2022)	Bangladesh	Landfill	CE ↑ CO_2_
Chew et al. (2022)	Malaysia	Pinch Analysis	CE ↑ CO_2_
Ma et al. (2023)	China	LCA	CE ↑ CO_2_
Hou et al. (2023)	Fly Ash	LCA	CE ↑ CO_2_

Note: ↑ increasing, ↓: decreasing, ≠: Insignificant, FGLS: Feasible Generalized least square, DPTM: Dynamic Panel Threshold Model, GMM: Generalized Method of Moments, PQR: Panel Quantile Regression, QQR: Quantile-on-Quantile Regression, STAR: Smooth Transition Autoregressive regression, MSV: Markov Switching Estimator, ARDL: Autorgression Distributive Lag model, GLS: Generalized Least Square, OLS: Ordinary Least Square, DOLS: Dynamic Ordinary Least Square, FMOLS: Fully Modified Ordinary Least Square, LCA: Life Cyle Assessment, CO2: Carbon emissions, CE: Circular economy, PP: Public-private Partnership, CP: Carbon Policy.

However, numerous drawbacks in the existing literature have been observed that must be incorporated to overcome the existing ambiguity. Firstly, the existing literature has not considered the panel data set to demonstrate the key problem and their best alternative for environmental sustainability. Therefore, the present empirical research considers specified economies due to three advantages, such as carbon policy, circular economy, and public-private partnership, to bring sustainability. Similarly, the existing literature has not tried to introduce a proper channel where economies contribute to environmental stress. For the first time, this study presents the fundamental problems and best alternatives to keep a clean & green environment under COP27. However, keeping in view that this would be the first empirical study that directly tries to investigate the significant role of green policy initiatives on environmental quality for selected economies, Interestingly, most studies have employed the direct connection of each variable with emissions; therefore, it would be an innovative step if this study introduces the mediating role of carbon policy, circular economy and PP on industrial sectors to evaluate indirect connection with emissions level. Finally, unlike the past literature, this study would try to utilize the most robust estimators to provide unbiased outcomes for concerned economies.

## 3 3- Data and methods

### 3.1 Data Collection and variables selection

This study introduces the critical environmental factors for well-organized economies from 2000 to 2021. However, these factors are the economic complexity index (ECI), population aging (PA), industrial development (ID), public-private partnership (PP) in energy, circular economy (CE), and carbon policy (CP). Discussing the key theme of each variable selection regarding environmental stress is imperative.

Under the theoretical justification, this study considers ECI as an alternative measure for development rather than focusing on income per capita. However, structural change has become a prominent phenomenon in the era of development and has received significant attention from policymakers. Therefore, the structural change can be expressed better via the ECI. However, for the first time, this theme was proposed by Ref. [48]. Similarly, this measure is being used in the current economy regarding changes in structure, qualities, know-how, and skills at the domestic level [49]. Therefore, it could be considered a leading environmental factor because it describes the most chic products and services [50]. Thus, it not only reflects the complex nature of the economy but also tries to cover the industrial structure [51]. Similarly, the population factor is also considered a determinant of CO_2_ emissions and could influence sustainability. Therefore, the present research utilizes population aging as the best proxy for the population to investigate its role in sustainability. However, this study considers an additional explanatory variable because a significant rise in productive population growth may be more responsible for environmental deterioration in the long run [52]. Also, it is an innovative variable that has not been used for sustainability, specifically in selected economies. Undoubtedly, the industrial sector has performed well in boosting the growth of economies, but it has also brought some negative externalities such as environmental stress. Therefore, the industrial sector has remained energy intensive sector that contributes to environmental stress [53]. Simply put, more industrial activities would bring more environmental deterioration; thus, it can be resolved via some green initiatives [54]. Furthermore, due to the rapid increase in industrial activities, the linear economy has become more prominent, and economies have utilized available resources in extensive ratios at zero environmental cost. Over time, used goods are transformed into garbage under the linear economy because no concept of reutilizing them exists. For instance, the linear economy is considered unsustainable due to its non-green behavior, which causes environmental stress. Therefore, the extensive ratio of wastage due to human activities would threat to biodiversity; consequently, economies have transformed their economies pattern from linear to circular economy (CE). The CE economy has become more prominent to offset the environmental harms and efficient allocation of scars resources. Thus, to boost the best reutilization of garbage, the economy transition is compulsory and its green spillover effects in term of waste reduction, reuse, and recycling could bring clean & green environment for forthcoming generations [55]. Similarly, the carbon policy also behaves well in managing environmental stress. Recently, carbon policy in the form of environmental taxes significantly reduces the level of emissions. However, it is common believe that the carbon policy would be more significant policy rather than energy tax. Also, carbon policy has remained a key factor that works well for sustainability under the Kyoto protocol and the Paris Agreement [56].

**Fig. 4:**
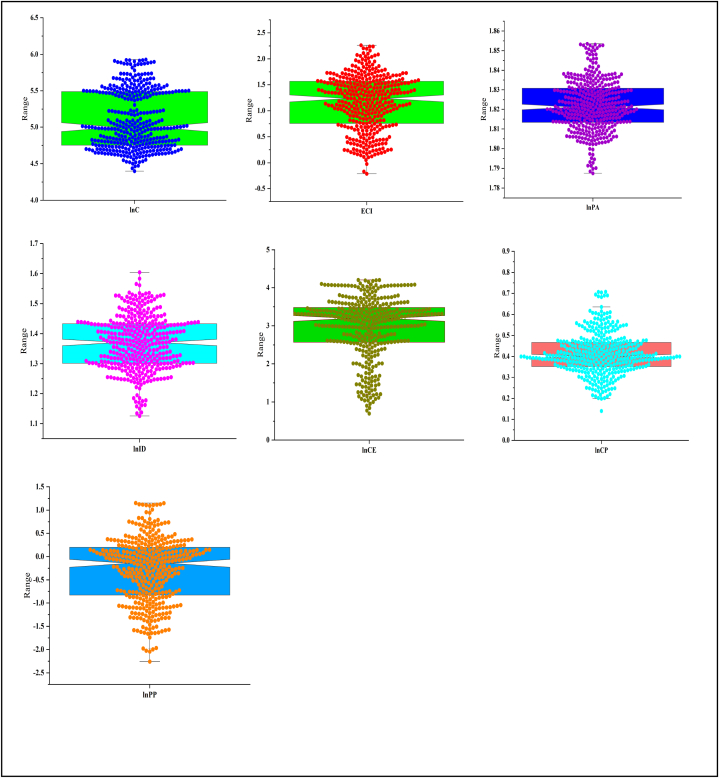
Box plots of the selected variable.

Similarly, Table 2 describes the key information regarding selected variables, such as units, symbols, and data sources. Simply put, the data for carbon emissions, industrial development, and population ages are taken from the World Development Indicators. Moreover, the public-private partnership in energy, economic complexity index, circular economy, and carbon policy are taken from different sources. However, all essential data information is given in Table 2 below. Similarly, Fig. 4 describes the bees’ plots of selected variables.

**Table 2:** Description of selected variables.

Symbol	Description	Unit	Source
Dependent Variable			
CO_2_	Carbon Emissions	Kt	WDI
Control Variables			
ECI	Economic Complexity	Index	Data Atlas
PA	Population aging (15–64 year)	% of total Population	WDI
Explanatory Variables			
ID	Industrial Development	Value added % of GDP	WDI
CE	Circular Economy	Energy recovery from wastage (Thousand Tonnes)	Eurostat
CP	Carbon Policy	Carbon Taxes (% of GDP)	Knoema
PP	Public Private Partnership	Billions of Constant 2011	IMF

### 3.2 Significance of study and selection of countries

**Fig. 5:**
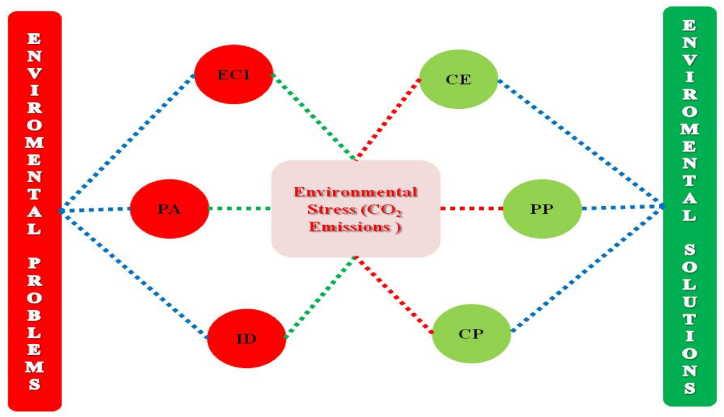
Conceptual framework of the study.

The present study has tried to convey a clear message regarding the problems and solutions to environmental sustainability under the ***COP27*** theme. This study aims to introduce an apparent depth regarding policy stability and government green initiatives to deal with rising environmental harms, especially for developed economies. For instance, this empirical research focuses on 17 developed economies to introduce the key problems and their best solution for environmental sustainability. Therefore, the present research would try to contribute to environmental economics via a memorable theme that has not been investigated. In the era of development, most nations have voluntarily designed development plans that only hit social & economic benefits, but they ignored the environmental challenges. Thus, this study is core to future research because it introduces the fundamental problems and their solution to combat environmental pollution. In these problems, this study highlights the contribution of the complex nature of the economy (ECI), demographic change (PA), and industrial progress (ID) towards environmental pollution. Similar to resolving environmental issues, it focuses on reutilizing garbage (CE), carbon policy (CP), and public-private partnerships. As per the best author's knowledge, this significant contribution to literature would guide policy analysts on the true meaning of green initiatives and their long-term effect on sustainability. However, in this era, economies have tried well to promote public-private investment in green projects for the sake of sustainable growth. However, the countries' selections were based on the following backgrounds: Firstly, these economies are considered top World economies with the potential to set policy targets, and countries across the globe have to follow such decisions. The leading example can be derived from the SDGs goals and targets that nations follow to become sustainable economies. Secondly, these countries have diversified tastes in their industrial activities and may have a divergent role in environmental deterioration. Therefore, selecting specified economies would offer a clear message as to whether these economies have attained a certain level in the industrial sector. Likewise, from the data perspective, selected countries have paid significant attention to green initiatives such as circular economy, carbon policy, and public-private partnerships to minimize environmental harm. Therefore, data availability is a leading factor in the selection of countries. Similarly, Fig. 5 describes the conceptual framework of the study that tries to clear the theme of this study.

### 3.3 Model construction and Hypothetical development

The current study introduces the key environmental factors such as the economic complicity index, population aging, industrial development, circular economy, carbon policy, and public-private partnership for the most well-organized economies globally. However, the given variables can be expressed in a single function.

(1) $$C_{i,t}=f\left(\gamma_{0},{ECI}_{i,t}^{\gamma_{1}},{PA}_{i,t}^{\gamma_{2}},{ID}_{i,t}^{\gamma_{3}},{CE}_{i,t}^{\gamma_{4}},{CP}_{i,t}^{\gamma_{5}},{PP}_{i,t}^{\gamma_{6}},\mu_{i,t}\right)$$

As per the function in equation (1), C, ECI, PA, ID, CE, CP, and PP expressed the carbon emissions, economic complexity index, population aging, industrial development, circular economy, carbon policy, and public-private partnership for the specified economies. However, the given function can be transformed into a log-log model via natural log on both sides.

(2) $${lnC}_{i,t}=\gamma_{0}+\gamma_{1}\;{ECI}_{i,t}+\gamma_{2}\;{lnPA}_{i,t}+\gamma_{3}\;{lnID}_{i,t}+\gamma_{4}\;{lnCE}_{i,t}+\gamma_{5}\;{lnCP}_{i,t}+\gamma_{6}\;{lnPP}_{i,t}+\mu_{i,t}$$

Similarly, Eq. (2) consists of lnC, lnPA, lnID, lnCE, lnCP, and lnPP, which refer to the natural log of carbon emissions, population aging, industrial development, circular economy, carbon policy and public-private partnership across the selected panel. Similarly, ECI refers to the economic complexity index used to measure progress development in selected economies. However, this study also describes some key hypotheses that must be described here. Firstly, in the era of development, most economies have faced a very complex nature of the entire economy, and they utilize available resources up to the optimum level. Thus, utilizing available resources only for rapid growth would bring environmental stress in the long run. Therefore, the present study imagines its coefficient would be positive for the environmental stress ($\gamma_{1}$ > 0). Similarly, population aging (range 15–64) is a key contributor to all human & economic activities; thus, it would not be an irrational comment that such a population could be productive population growth. However, the utilization of energy and other human activities would bring environmental stress at the domestic level, and its coefficient would be positive ($\gamma_{2}$ > 0). More interestingly, the rapid growth has remained an essential function of industrial activities that are also well connected with climate change. Thus, it is imperative to make clear that most nations have tried well to make the industrial transition from emission-intensive to eco-friendly industries. However, this sector has not been adequately managed to maintain the environmental standards under the green theme. Therefore, the massive utilization and extraction of resources for the sake of industrial development could bring massive pollution to the atmosphere ($\gamma_{3}$ > 0). Since the last three decades, reutilizing used products under the circular economy (CE) has become attractive for higher authorities to deal with biodiversity losses. Therefore, the present era has focused on generating energy from garbage or other disposed material to keep clean & green; due to such green initiatives, the present study imagines that its coefficient would be damaging over time ($\gamma_{4}$ < 0). Carbon policy is another crucial phenomenon in dealing with environmental stress, and most nations have tried to implement it properly. In this regard, carbon trading, energy taxes, and carbon taxes have been introduced, but to this date, carbon taxes become a more efficient tool to cover climate change. It is a common belief that selected economies have settled their ambition to control rising emissions via introducing carbon policy, and its coefficient would be negative ($\gamma_{5}$ < 0). Finally, the public-private partnership has also been considered the best alternative to proceed with a green environment. Therefore, economic actors (government or consumers) can't deal with all issues alone; for example, to deal with environmental problems, the UN has proposed Goal 17 to ensure the public-private partnership (PP) for sustainable growth. By having a key image regarding PP, its coefficient would be harmful to leading the world's economies ($\gamma_{6}$ < 0).

Besides, industrial activities cannot be stopped to resolve environmental issues, but they can be managed. Therefore, the present empirical research tries to introduce the mediating effect of public-private partnership, carbon policy, and circular economy on industrial activities to check out the response of each variable to the sustainability theme. Under the mediation role of public-private partnership on industrial development ln(PP*ID), this study imagines that the PP has not fully paid attention to green industrial activities. Consequently, the PP at the domestic level may only perform as a symbol of cooperation, but its effect could be meaningless. Its coefficient would be insignificant in the long run, and the model can be written as,

(3) $${lnC}_{i,t}=\gamma_{0}+\gamma_{1}\;{ECI}_{i,t}+\gamma_{2}\;{lnPA}_{i,t}+\gamma_{3}\;{lnID}_{i,t}+\gamma_{4}\;{lnCE}_{i,t}+\gamma_{5}\;{lnCP}_{i,t}+\gamma_{6}\;{lnPP}_{i,t}+\gamma_{7}\;{\ln\;\left(PP*ID\right)}_{i,t}+\mu_{i,t}$$

Eq. (3) shows ln(PP*ID) as the mediating role of PP on industrial activities. Similarly, carbon policy (CP) cannot be excluded from this race, and the present study also offers CP's mediating effect on industrial development (See Eq. (4)). Extensive taxes on emissions-intensive activities could stop them. Therefore, industrial activities secure the environment by reducing emissions and trying to manage industrial garbage. This slope could be harmful to selected countries in the long run, and the mediating model can be expressed as,

(4) $${lnC}_{i,t}=\gamma_{0}+\gamma_{1}\;{ECI}_{i,t}+\gamma_{2}\;{lnPA}_{i,t}+\gamma_{3}\;{lnID}_{i,t}+\gamma_{4}\;{lnCE}_{i,t}+\gamma_{5}\;{lnCP}_{i,t}+\gamma_{6}\;{lnPP}_{i,t}+\gamma_{7}\;{\ln\;\left(CP*ID\right)}_{i,t}+\mu_{i,t}$$

Finally, equation (5) exhibits the circular economy (CE) mediation effect on industrial development. Covering the industrial and other economic sectors and ensuring raw material reuse for energy generation perspectives would be the better strategy. Therefore, its coefficient would be negative for environmental stress ($\gamma_{7}$ < 0).

(5) $${lnC}_{i,t}=\gamma_{0}+\gamma_{1}\;{ECI}_{i,t}+\gamma_{2}\;{lnPA}_{i,t}+\gamma_{3}\;{lnID}_{i,t}+\gamma_{4}\;{lnCE}_{i,t}+\gamma_{5}\;{lnCP}_{i,t}+\gamma_{6}\;{lnPP}_{i,t}+\gamma_{7}\;{\ln\;\left(CE*ID\right)}_{i,t}+\mu_{i,t}$$

However, this study also suggests some key hypotheses to understand the theme under the specified model construction.

H1 Economic complexity index positively contributes to environmental stress.

H2 Population aging positively contributes to environmental stress.

H3 Industrial development enhances the environmental stress.

H 4Circular economy contributes to environmental quality.

H5 Carbon Policy significantly enhances the environmental quality.

H6 Public-private Partnerships increase the environmental quality.

### 3.4 Estimation strategy

The present empirical study tries to utilize the most reliable estimators that deal with panel data problems and bring reliable outcomes. Thus, due to globalization, economies are interconnected, and there may be a chance for cross-sectional dependence in the selected data. For illustration, this study utilizes the four CSD tests to obtain the reliable outcomes proposed by Refs. [[57], [58], [59], [60]]. Similarly, under the CSD, this study performs the slope of homogeneity test as suggested by Ref. [61]. Interestingly, the traditional data integration tests do not perform under the CSD; therefore, this study considers the advanced tests, i.e., CADF & CIPS [62]. However, both integration tests cannot demonstrate the structural break across the panel data; we utilize Lee and Strazicich (Lee and Strazicich, 2003) to overcome this issue. Likewise, to investigate the long-term cointegration among selected variables, this study performs the advanced cointegration test as suggested by Ref. [63]. Finally, with a significant data validation series, the present research moves toward the long-run estimator to obtain the study objectives.

Different estimators have been introduced recently to investigate the study objectives, but the mean group series has become more prominent. Therefore, the present study utilizes the Augmented Mean Group (AMG) estimator for robust and reliable outcomes [64,65]. This estimator has the ability to perform well in the presence of CSD and heterogeneity. However, there has been a minor difference between the AMG and common Correlated Effect Mean Group (CCE-MG) estimators that can be understood via unobserved common factors. The CCE-MG estimator consists of observed common factors and the cross-sectional average of dependent & independent variables [66]. However, there are two different steps where AMG can predict unobserved common dynamic effects and CSD. Firstly, it demonstrates Eq. (6) with a dummy variable and tries to estimate the first difference in OLS.

(6) $$\Delta y_{it}=\alpha_{1i}+\beta_{i}{\Delta x}_{it}+\phi_{i}f_{t}+\sum_{t=2}^{T}\tau_{t}{DUMMY}_{t}+\varepsilon_{it}$$

Similarly, τ refers to a common dynamic process under the AMG estimator [67]. However, Eq. (7) describes the general form of AMG estimator,

(7) $$AMG=N^{-1}\sum_{i=1}^{N}{\tilde{\beta }}_{i}$$

Whereas in Eq. (8), ${\tilde{\beta }}_{i}$ is being considered as the estimate of the coefficient.

(8) $$\Delta y_{it}={\alpha_{1i}+\beta }_{i}{\Delta x}_{it}+\phi_{i}f_{t}+\sum_{t=2}^{T}\tau_{t}{DUMMY}_{t}+\varepsilon_{it}$$

**Fig. 6:**
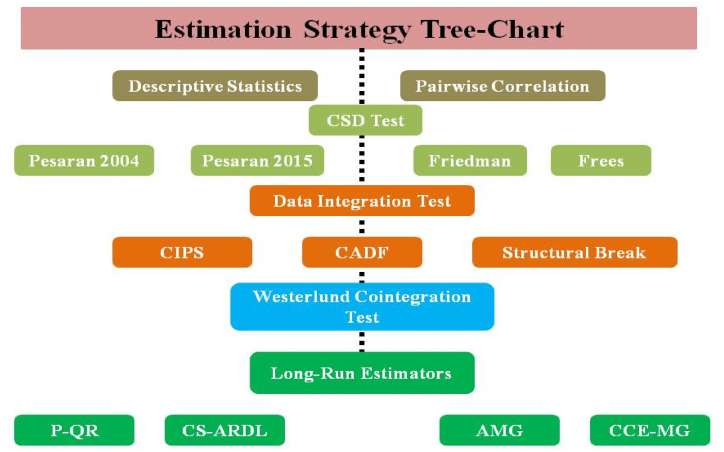
Chart flow for estimation strategy.

Moreover, this empirical research utilizes the CCE-MG as a robust estimator to revalidate the outcomes by the AMG estimator as proposed by Refs. [66,68]. Similarly, for the specified economies, the present study does not believe in few estimator outcomes; consequently, this study also utilizes the Cross-Sectional Autoregressive Distributive Lag model (CS-ARDL) for further validation of investigated outcomes by AMG & CCE-MG estimators, and this estimator has been suggested by Ref. [69]. However, numerous case studies have utilized such estimators to investigate their study objectives (refers: [[70], [71], [72]]). Similarly, Fig. 6 describes the flow chart of the estimation strategy for a quick review.

## 4 4- Results and discussion

This section provides detailed empirical outcomes and variables responding to the specified economies' environmental stress. However, before moving forward to long-run association among selected variables, the present research offers some data validation tests. However, descriptive statistics and pairwise correlation tests are considered to be the initial screening of the selected data. Similarly, Table 3 describes the outcomes of the pairwise correlation test and validates the prior expectations of no outlier in panel data. As per given outcomes, there is no large difference between the mean & median values of the selected variables that strongly supports no presence of outlier in the provided data.

**Table 3:** Descriptive statistics.

	lnC	ECI	lnPA	lnID	lnCE	lnCT	lnPP
Mean	5.1066	1.1645	1.8221	1.3687	2.9650	0.4124	−0.2909
Median	5.0003	1.2366	1.8212	1.3708	3.1947	0.4005	−0.1441
Maximum	5.9282	2.2616	1.8534	1.6043	4.2035	0.7075	1.1519
Minimum	4.3982	−0.2103	1.7874	1.1255	0.6989	0.1398	−2.2568
Std. Dev.	0.4005	0.5324	0.0131	0.0906	0.8146	0.0987	0.7042
Skewness	0.3839	−0.3376	0.1005	−0.0694	−0.8367	0.4213	−0.3323
Kurtosis	1.8723	2.3214	2.9046	2.6124	3.0246	3.4722	2.5068

Table 4 describes the outcomes regarding the pairwise correlation test, showing that no single variable has more than a 0.80 % correlation value. From such interesting outcomes, this study exhibits no multicollinearity observed in the existing data. However, this study also performs the VIF test to revalidate the presence of multicollinearity.

**Table 4:** Correlation analysis.

Correlation	lnC	ECI	lnPA	lnID	lnCE	lnCT	lnPP	VIF	1/VIF
lnC	1.000							–	–
ECI	0.4110	1.000						2.14	0.4672
lnPA	0.0224	−0.0575	1.000					1.99	0.5019
lnID	−0.0202	0.3125	0.6329	1.000				2.93	0.3408
lnCE	0.5137	0.5626	−0.3845	−0.1959	1.000			2.08	0.4802
lnCT	−0.3549	−0.0204	−0.1279	−0.2445	−0.0449	1.000		1.48	0.6735
lnPP	0.4452	−0.1099	−0.2014	−0.3865	0.2485	−0.3682	1.000	1.70	0.5882
Mean VIF								2.06	

Table 5 describes the outcomes of CSD and the slope of homogeneity tests. Simply put, Table 4 is divided into two parts, lower and upper; therefore, the upper panel describes the result of CSD tests and validates our prior expectations. Moreover, the lower panel of Table 4 shows the outcome related to the slope of homogeneity. However, the results given are as per the study's prior expectations.

**Table 5:** CSDs and Slope of Homogeneity tests.

Variable	Pesaran (2004)	Pesaran (2004)	Frees' test	Friedman's
lnC	46.486***	46.486***	10.424***	287.804***
ECI	8.994***	8.994***	5.286***	64.067***
lnPA	49.010***	49.010***	10.977***	296.068***
lnID	26.503***	26.503***	5.826***	173.870***
lnCE	28.158***	28.158***	8.161***	194.053***
lnCT	8.410***	8.410***	1.972***	61.196***
lnPP	5.465***	5.465***	2.417***	43.276***
Slope of Homogeneity test				
Statistics	Delta		Adjusted	
	8.301***		10.167***	
LM	334.4***		0.000	
LM adj*	17.82 ***		0.000	
LM CD*	15.08 ***		0.000	

Note: *** shows the significance level at 1 %.

Similarly, Table 6 describes the outcomes of data integration by CADF & CIPS tests. However, as per the given outcomes, all selected variables are integrated at the first difference, except for circular economy (CE) and public-private partnership (PP) for selected economies. However, the structural break in the panel data cannot be investigated by the CADF & CIPS unit root test.

**Table 6:** Outcomes of Integration test.

	CADF		CIPS	
	Level	1st Difference	Level	1st Difference
lnC	–	−4.606***	–	−4.625***
ECI	–	−4.118***	–	−4.120***
lnPA	–	−3.592***	–	−3.511***
lnID	–	−4.275***	–	−4.270***
lnCE	−2.172**	–	−2.170**	–
lnCT	–	−4.235***	–	−4.235***
lnPP	−2.163**	–	−2.163**	–

Note: *** and ** show the significance level at 1 % and 5 %, respectively.

Similarly, Table 7 describes the structural break unit root test and the exciting outcomes. As per given outcomes, selected variables are integrated at different significance levels. Also, the outcomes showed that all integrated variables have a structural break and CSD properties.

**Table 7:** Structural Break Unit Root test.

	Constant			Trend		
	Value	Break	CSD	Value	Break	CSD
lnC	−11.7064***	1	Yes	−7.7491***	1	Yes
ECI	−3.0438**	1	Yes	−4.3163***	1	Yes
lnPA	−12.2756***	1	Yes	−4.1619***	1	Yes
lnID	−8.8633***	1	Yes	−2.2239**	1	Yes
lnCE	−6.0948***	1	Yes	−3.1958***	1	Yes
lnCT	−1.9394**	1	Yes	−3.7418***	1	Yes
lnPP	−5.1141***	1	Yes	−3.9393***	1	Yes

Note: ***, and ** show the significance level at 1 %, and 5 %, respectively.

However, before the long-run cointegration among the selected variables, this study cannot perform any estimators to investigate the deep connection among selected variables. Thus, per the given conditions, this study obtains strong evidence regarding cointegration among selected variables and rejects the null hypothesis of no cointegration (see Table 8).

**Table 8:** Long-run Cointegration test.

Statistics	Value	Z-value	P-value	Robust P-value
Gt	−1.883	5.729	1.000	0.000
Ga	−2.845	7.826	1.000	1.000
Pt	−6.614	5.593	1.000	0.000
Pa	−2.893	6.371	1.000	0.000

### 4.1 Long run outcomes by augmented mean group (AMG)

The present empirical research investigates the long-run connection of selected environmental determinants with emissions levels in selected economies and utilizes the augmented mean group (AMG) estimator for the base model (see Table 9). Along with the AMG estimator, the present research compares AMG outcomes with two well-robust estimators, CCE-MG and CS-ARDL. For instance, the economic complexity index (ECI) is the first determinant of environmental stress. Given the outcomes, any significant change in this factor would cause an increase in emissions of 0.014 % under the AMG specification. On the other hand, the outcomes by CCE-MG & CS-ARDL estimators do not vary from the prior investigation, and ECI positively contributes to environmental damages by 0.001 % and 0.024 %, respectively. Thus, the results of ECI represent that the complex nature of the economy is not well suited for selected economies. However, this connection must be explained with some key logic to understand the precise situation of selected economies. Under the general arguments, because of the complex nature of the economy, most nations are directly associated with energy-intensive activities via the industrial, services, Agri, and mining sectors. Over time, there has been an increase in production activities, and economies have made significant progress to ensure green activities, but these have not reached sufficient conditions. Therefore, the efficient utilization of available resources in terms of advanced methods, technologies, and efficient labor has not met the minimum level of a green environment, and emission problems have increased over time. Similarly, excessive demand for products and their production process demands massive energy consumption; consequently, it raises environmental harm at the domestic level. Also, the tradeoff between the ECI and ecological quality could be due to continuous fluctuation in the balance of payment under the open economy. Simply put, there has been an entire connection between trade activities and ECI, directly raising emissions levels. Simultaneously, it is impossible to meet the products' quality & quality at the minimum standard of sustainability across all sectors; thus, due to the complex nature of products and their energy-intensive behavior causes environmental stress. Undoubtedly, the specified economies have made their best efforts to adopt the green measures under the SDGs, but emissions are continuously rising. However, the behavior of the populace in the domestic economy also causes environmental deterioration. With a significant rise in income and job opportunities in the open economy, people try to utilize their income share on luxury items, and most have energy-intensive behavior. Consequently, there has been a rising trend in emissions levels over time. This outcome is coherent with the findings of prior studies such as [50,73,74]. Therefore, discussing the comparative arguments suggested by the relevant studies mentioned above is necessary. As per the given arguments Neagu and Teodoru [50], most nations have complex natures regarding their import, export, and other sectors of the economy that indirectly influence the environmental quality at the domestic level. Thus, an immense dependence on energy-intensive activities such as transport and inefficient infrastructure would bring ecological stress along with the complex nature of imports & exports of products.

**Table 9:** Outcomes for the base model.

	AMG		CCE-MG		CS-ARDL	
	Coeff.	Std. Error	Coeff.	Std. Error	Coeff.	Std. Error
ECI	0.0149**	0.0222	0.0011**	0.0257	0.0244***	0.0262
lnPA	0.1774***	1.5337	5.7774**	3.4466	0.3551***	1.7084
lnID	0.0797**	0.1122	0.0679***	0.1831	0.0299***	0.2189
lnCE	−0.0167**	0.0172	−0.0022***	0.0278	−0.0196***	0.0568
lnCP	−0.0112**	0.0480	−0.0779**	0.0793	−0.3084**	0.1017
lnPP	−0.0025***	0.0049	−0.0045***	0.0118	−0.0083**	0.0061
Cons.	−4.7786***	2.7934	−2.3465**	3.9444	–	–
					Short Run CS-ARDL	
${lnC}_{t-1}$	–	–	–	–	−0.5813**	0.3807
ECI	–	–	–	–	0.0118**	0.0251
lnPA	–	–	–	–	0.7984*	1.8814
lnID	–	–	–	–	0.1093*	0.2148
lnCE	–	–	–	–	−0.0254**	0.0581
lnCP	–	–	–	–	−0.3141***	0.1019
lnPP	–	–	–	–	−0.0072*	0.0069

Note: ***, **, and * are significant at 1 %, 5 %, and 10, respectively.

Interestingly, the given coefficient value of population aging (PA) describes the positive connection with environmental stress. Simply put, any significant change in this factor would cause an increase in emissions levels by 0.177 % under the AMG specification. However, this connection is also validated by the CS-ARDL & CCE-MG estimators, which refer to a 1 % rise in PA, which would bring environmental stress by 5.777 % and 0.355 %, respectively. All estimators strongly support the positive association among selected variables; thus, discussing some core arguments on their behalf is necessary. Considering that the population aging consists of a population aged from 15 years to 64 years, the specified connection must be explained via different opinions. Firstly, most nations have tried to increase the quality of population growth via rapid progress in the education sector, but these economies have not significantly paid attention to its negative externalities in terms of emissions. Consequently, due to the rise in educational activities, the populace utilizes energy-intensive products that emit pollutants into the atmosphere and cause greenhouse gas emissions. Secondly, a significant increase in income level leads to a better standard of living. However, it has been observed that the consumption pattern of specified age groups differs from children and older people. Such variation in lifestyle and consumption patterns would increase the consumption of available resources and cause pollution. Therefore, the rapid growth in income per capita is also a phenomenon behind the PA's connection with environmental stress. Thirdly, it is a common belief that with a significant increase in population age, every person has tried to connect with their family in each matter of life, for example, over time people try to live with their children and travel together for food, shopping, functions, etc. Consequently, a significant rise in energy-intensive activities may bring massive environmental stress and affect humankind. A substantial increase in labor activities for economic activities such as industrial, services, and transport sectors also boosts emissions-intensive activities and causes environmental harm. However, the present outcome is also in line with the findings of [75,76]. Similarly, these supportive studies also have interesting arguments regarding this connection. For example, Wang and Wang [77] described their association of PA under the theme of industrial structure and environmental quality. Therefore, the threshold model explains that population aging causes less ecological harm at the initial stages of industrial development but causes environmental stress at later stages. Moreover, Zhou et al. [78], described population aging has a more complex nature with environmental quality. They investigated the quadratic form of PA with CO_2_ emissions and found the U-shaped & inverted U-shaped association between the concerned variables in different Chinese provinces. Therefore, the PA structure is highly complex and can vary from province to province.

Since the Industrial Revolution, economies have significantly boosted their development progress at any cost of environmental damage. Therefore, the present study tries to investigate the role of industrial development in sustainability for the selected economies, and it shows a positive impact on the level of emissions. In simple words, a 1 % rise in this factor would cause an increase in emissions level by 0.079 %, under the AMG estimator. Similarly, the CCE-MG and CS-ARDL estimators validate the AMG outcome and describe any significant change in this factor that may cause environmental stress by 0.067 % and 0.029 %, respectively. However, over time, policymakers commonly believe that heavy industries and pollution-intensive sectors may consume massive energy resources to run their production activities; consequently, emissions rise. Similarly, most industries are in a transition phase; either these are transforming their infrastructure from primary to secondary or secondary to tertiary. Across the globe, a common phenomenon has been observed: the share of secondary industries is greater than that of tertiary industries, which may cause long-term environmental stress. However, for rapid growth, economies have tried to transform their entire economic structure from labor-intensive to knowledge-intensive, which may increase sustainability. In reality, a rise in knowledge would produce the same outcome without green initiatives such as adopting green technology, renewable energy consumption, and green production methods. Thus, it would not be an irrational comment that specified economies rely on the secondary industrial setup that emits massive pollution in production activities due to energy-intensive behavior. Also, this outcome supports the findings of [[79], [80], [81]]. Interestingly, Dong et al. [80], showed the counterarguments in the case of China and described the inverse connection between IND and environmental pollution. However, the authors have explained that this behavior has resulted from joining China's economy to the World Trade Organization, which intensively supports resource conservation and a green environment under the industrial upgrade. Similarly, Hu and Man [82] supported the positive association but did not provide straightforward arguments for the investigated outcomes.

In recent years, the circular economy has received immense attention from policy analysts to reuse the resources used for different purposes, such as energy generation. Therefore, this study also considers circular economy (CE) as a green measure of sustainability, showing fascinating outcomes. For example, a 1 % rise in this factor (CE) would cause a decline in emissions by 0.016 % at a 5 % significant level. Also, this connection validates by CCE-MG & CS-ARDL that any circular economy fluctuation would help reduce emissions by 0.002 % and 0.019 %, respectively. Therefore, the rising trend in greenhouse gas emissions due to massive wastage by human & economic activities has become a serious threat to humankind. In order to deal with such biodiversity loss, well-organized economies have taken the green initiative to reutilize such wastage into energy generation. From this step, the rapid energy thirst can be minimized at a minimum bio-diversity loss. In general, to deal with rising environmental stress, the circular economy is considered an essential factor in the era of development [83]. However, CE does not only protect environmental quality but also promotes resource conservation and sustainable growth. Simply put, the rapidly industrialized sector polluted the environment via direct (carbon pollution) & in-direct (industrial wastage) ways, and successful implementation of CE could be a last resort [84]. Undoubtedly, the best utilization of wastage could be possible when economies have an excellent technological setup that helps to reutilize green energy and protect the environment. From such an exciting outcome, it is clear that the specified economies have achieved a certain technological level and are on the right path to secure their biodiversity. Similarly, this outcome can compare with the findings of [[85], [86], [87]]. Similarly, Khan et al. [86], described the case study of Netherlands and argued that higher authorities had taken the initiative to utilize waste in green energy generation and other sustainable production & consumption activities at the domestic level. Such interesting initiatives may bring less environmental stress in the near future.

Similarly, carbon policy (CP) is also considered a green initiative to deal with environmental problems. As per the given outcomes by the AMG estimator, any significant change in carbon policy would cause a decline in emissions by 0.011 % for the selected panel. Similarly, a 1 % rise in carbon policy also brings the clean environment by 0.077 % and 0.308 %, respectively, under the CCE-MG & CS-ARDL estimators. Undoubtedly, in the last few decades, the higher authorities have compelled all stakeholders to follow the environmental standards; otherwise, you have to pay heavy duties on emissions-intensive activities. Numerous initiatives have been proposed, such as carbon trading, energy tax, and energy subsidies, but carbon policy in the form of taxes has become more significant. Undoubtedly, the carbon policy works well to manage environmental stress, but it is associated with a clean environment in two different ways. Firstly, a significant rise in the carbon tax would decrease the consumption of energy-intensive products and compel people to use energy-efficient products that ultimately bring environmental quality. Secondly, a substantial increase in carbon policy may discourage industrial production activities that decline the environmental pressure, along with economic progress. Therefore, it is necessary to ensure a sustainable environment without any social and economic loss at the domestic level. Such outcomes are occupied with the findings of [25,88,89]. However, Bashir et al. [25], described the key role of environmental policy in OECD economies and found the supportive role of environmental tax in environmental quality. Moreover, they suggested allocating environmental tax revenue efficiently to minimize environmental stress over the long run.

Finally, the public-private partnership (PP) also describes the inverse connection with emissions level. However, this trend has been observed recently, and policymakers are trying to compel economic actors to collaborate and fight for long-term sustainability. Thus, the selected economies have made significant progress in PP, and this study utilizes it as an environmental factor. Simply put, it describes the negative connection with emissions levels. A 1 % rise in this factor would cause a decline in environmental stress by 0.002 %, 0.004 %, and 0.008 %, respectively, under the mentioned specifications. Generally, rising environmental stress has become a key challenge at the forefront of policy analysts, and they are endorsed World with different practices such as PP at the domestic level. Thus, at this time, selected nations and whole economies across the globe are trying to make economic transformation that is impossible without green energy, technical innovations, efficient utilization of resources, and sustainable progress. For instance, the UN has proposed SDG 17 to promote public-private partnerships in different aspects of life. This initiative aims to protect future generations from environmental harm. If specified economies have settled their ambition to become clean & green by 2050, they must follow PP's key because a single effort by higher authorities may not play well. Interestingly, the dream for green initiatives and their spillover effect cannot be achieved without collaboration. However, the current outcome aligns with the findings of recent case studies [90,91]. Also, Anwar et al. [92], described the case study of China to investigate the role of PP in environmental sustainability and found supportive outcomes. In order to maximize public-private partnerships, the government has brought significant initiatives in the transport sector that increased green energy share and reduced environmental stress.

Similarly, the lower panel of Table 9 describes the short-run outcomes obtained by the CS-ARDL estimator. The control variables of the study (ECI & population aging) are investigated to find if they have a positive connection with emissions levels. Similarly, from the explanatory variables, industrial development has a positive but insignificant impact on CO_2_ emissions in the short run. Similarly, the circular economy & carbon pricing significantly reduces the environmental pressure for specified economies. However, this behavior has been observed for public-private partnerships but remains insignificant in the short run. The given value of cointegration (−0.581 %) describes the convergence speed from the short to the long run. Thus, it will require less than half a year to converge in selected economies in the short-to long-term.

### 4.2 The mediating effect outcomes by AMG estimator

In this era of development, the industrial sector has made significant contributions to development plans, but it has also been harming environmental quality. However, Table 10 describes the four different models of the study and finds exciting outcomes. As per the given outcomes, the industrial sector positively contributes to environmental damages by 0.079 %, 0.006 %, 0.114 %, and 0.056 %, respectively, across all models. These outcomes indicate that the industrial sector presently exhibits energy-intensive behavior, contributing to long-term environmental stress. Thus, due to its harmful behavior toward sustainability, the present study also tries to evaluate the mediating role of key solutions, i.e., public-private partnership, carbon policy & circular economy on industrial development. Similarly, column 2 describes the mediating role of public-private partnership on industrial development [lnPID(lnPP*lnID)], and it shows the positive but insignificant role in rising emissions. It infers that any fluctuation in public-private partnerships for industrial activities has no significant contribution; consequently, it causes emissions in the long run. This behavior has been observed due to the personal interest of all stakeholders. For example, the business sector aims to earn a profit, and business leaders are not concerned about environmental sustainability. In contrast, the government prioritizes collecting taxes from energy or emission-intensive industries to prolong their development plans. Consequently, there has been a mismatch between the priorities of stakeholders, and emissions are rising. Therefore, to keep green societies at the domestic level, all stakeholders must understand the current demand for green environments to manage environmental problems with collective actions. In the policy perspective theme, specified countries should care for their environmental quality, and PP should be promoted equally across all sectors.

**Table 10:** Mediating effect outcomes by AMG estimator.

Variable	Column 1	Column 2	Column 3	Column 4
	lnID	lnPID	lnCID	lnCEID
lnID	0.0797**	0.0065*	0.1140**	0.0561**
	0.1122	0.1565	0.1291	0.1186
lnPID	–	0.1577*	–	–
	–	0.1667	–	–
lnCID	–	–	−0.0580***	–
	–	–	0.0734	–
lnCEID	–	–	–	−0.0089***
	–	–	–	0.0150
ECI	0.0149**	0.0166***	0.01745**	0.0325**
	0.0222	0.0195	0.0208	0.0179
lnPA	0.1774***	0.4131**	0.6833***	0.9652***
	1.5337	1.7564	1.1850	1.6407
lnCE	−0.0167**	−0.0102**	−0.0077***	−0.02155***
	0.0172	0.0162	0.01704	0.0223
lnCP	−0.0112**	−0.0035**	−0.0756**	−0.0032**
	0.0480	0.0556	0.0708	0.0587
lnPP	−0.0025***	−0.2341***	−0.0424**	−0.0206***
	0.0049	0.2364	0.0439	0.0303
Cons.	−4.7786***	−4.5696***	−4.3269***	−3.4661**
	2.7934	3.1876	2.3622	2.9958

Note: ***, **, and, * show the significance level at 1 %, 5 %, and 10, respectively.

Moreover, column 3 describes the mediating effect of carbon policy on industrial development [lnCIP(lnCP*lnID)] and the given coefficient value of the mediating role (−0.058) at a 1 % significance level. It refers to any significant change in this mediating effect that would cause to reduce environmental stress by 0.058 % for concerned economies. Notably, carbon policy can deal with environmental harm due to the industrial sector. Its coefficient is minor because this study considers only the industrial sector as a mediator for carbon policy. From this outcome, it is understood that carbon policy works well to manage environmental stress at the domestic level, and selected economies are on the right path. Finally, the mediating role of the circular economy also plays a significant role in controlling environmental deterioration. It refers to any significant change in this factor (lnCEID) that would cause a decline in emissions by 0.008 % for the specified economies. The carbon policy is undoubtedly attractive but cannot control biodiversity loss via excessive industrial garbage. Therefore, to overcome biodiversity loss, countries have relied on a circular economy that helps reuse the trash and convert it into energy inputs. More interestingly, it secures the environment and tries to reduce over-dependency on traditional energy that may further cause environmental pollution. However, by keeping everything constant, the economic complexity index and population aging are positively associated with environmental stress. Also, the leading roles of public-private partnership, circular economy, and carbon policy do not deviate from the prior outcomes.

### 4.3 Mediating Robustness analysis

Table 11 describes the outcomes investigated by the CCE-MG estimator, and this study finds the most reliable outcomes that support prior outcomes by the AMG estimator. As per the given outcomes, the control variables such as ECI & population aging significantly deteriorate sustainability levels in the specified economies. Moreover, industrial development substantially contributes to environmental degradation across all models. Finally, the present study examines the role of CP, circular economy, and public-private partnership in environmental quality for specified economies. However, the mediating effect of carbon policy, circular economy, and PP on industrial development offers some interesting findings. Similarly, column 2 consists of public-private partnership mediating impact on industrial development, and it describes the positive but insignificant role in environmental pollution. Interestingly, column 3 presents the mediating role of carbon policy on industrial development and reduces environmental deterioration by 0.366 %. Finally, column 4 exhibits the negative connection between CE's mediation role on industrial activities and how it diminishes ecological stress in the long run.

**Table 11:** Mediating Robustness analysis by CCE-MG estimator.

	Column 1	Column 2	Column 3	Column 4
Variable	lnID	lnPID	lnCID	lnCEID
lnID	0.0797**	0.1188***	0.2083**	0.0565
	0.1122	0.2715	0.1719	0.2086
lnPID	–	0.0265*	–	–
	–	0.4825		
lnCID	–	–	−0.3662***	–
	–		0.3489	
lnCEID	–	–	–	−0.0339***
	–			0.0257
ECI	0.0149**	0.0095**	0.0007***	0.0096***
	0.0222	0.0375	0.0256	0.0334
lnPA	0.1774***	6.3892**	7.7109**	7.1194**
	1.5337	3.5998	4.2967	3.4533
lnCE	−0.0167**	−0.0048**	−0.0289***	−0.0113**
	0.0172	0.0315	0.0295	0.0269
lnCP	−0.0112**	−0.1315***	−0.2811**	−0.0788***
	0.0480	0.0936	0.4497	0.0813
lnPP	−0.0025***	−0.0147**	−0.2505**	−0.0737**
	0.0049	0.6693	0.2166	0.0495
Cons.	−4.7786***	−3.5718**	−0.1287***	−1.5829***
	2.7934	4.5073	5.4482	4.6511

Note: ***, **, and * are significant at 1 %, 5 %, and 10, respectively.

Similarly, Table 12 exhibits the key outcomes obtained by the CS-ARDL estimator for the selected panel data set. By keeping all things constant, the mediating role of PP on industrial development shows a positive but insignificant impact on environmental sustainability. Moreover, the mediating role of CE and carbon policy on ID evaluates the negative role of environmental pollution. Therefore, both initiatives (CE & CP) significantly reduce the emissions level via the industrial sector. Under the short run, the cointegration value describes the speed of adjustment from the short to the long run. As per the given values, the selected economies may take less than half a year to converge from the short to the long run.

**Table 12:** Mediating Robustness analysis by CS-ARDL estimator.

Variables	Column 1	Column 2	Column 3	Column 4
	lnID	lnPID	lnCID	lnCEID
lnID	0.1093*	0.2373*	0.4218**	0.1506**
	0.2148	0.3010	0.3865	0.1939
lnPID	–	1.0692***	–	–
	–	0.3489	–	–
lnCID	–	–	−0.6578**	–
	–	–	0.5971	–
lnCEID	–	–	–	−0.0283**
	–	–	–	0.0657
ECI	0.0118**	0.0081**	0.0279**	0.02188*
	0.0251	0.0265	0.0296	0.03001
lnPA	0.7984*	0.5515***	0.4936**	0.7747*
	1.8814	2.0789	2.1142	2.7545
lnCE	−0.0254**	−0.0277**	−0.0363**	−0.0236*
	0.0581	0.0591	0.0496	0.0512
lnCT	−0.3141***	−0.3038***	−1.2337***	−0.3414**
	0.1019	0.1001	0.8928	0.1642
lnP	−0.0072*	−1.4842***	−0.4149	−0.0642**
	0.0069	0.4976	0.3807	0.1172
${lnC}_{t-1}$	−0.5813**	−0.6691**	−0.6962***	−0.6585***
	0.3807	0.0851	0.0891	0.1061
Long-Run Outcomes by CS-ARDL				
lnID	0.0299***	0.0748**	0.2716**	0.1005**
	0.2189	0.2528	0.2865	0.2283
lnPID	–	0.9392*	–	–
	–	0.3685	–	–
lnCID	–	–	−0.5524***	–
	–	–	0.3901	–
lnCEID	–	–	–	−0.1525**
	–	–	–	0.1241
ECI	0.0244***	0.0119***	0.0411**	0.0583**
	0.0262	0.0214	0.0375	0.0422
lnPA	0.3551***	−0.8277***	0.4262**	1.6015***
	1.7084	1.8577	2.2362	2.4633
lnCE	−0.0196***	−0.0556***	−0.0224**	−0.0134***
	0.0568	0.0786	0.0452	0.0529
lnCP	−0.3084**	0.3041***	0.8298***	−0.3454**
	0.1017	0.1079	0.5364	0.1681
lnPP	−0.0083**	−1.2961**	−0.3197**	−0.2924**
	0.0061	0.5211	0.2341	0.2475

Note: ***, **, and, * show the significance level at 1 %, 5 %, and 10, respectively.

### 4.4 Heterogeneity analysis by panel quantile regression

Table 13 describes the key outcomes by Panel Quantile regression (P-QR). However, the obtained outcomes don't deviate from the prior outcomes obtained by the mentioned estimators. Simply put, Table 13 describes each variable's response toward carbon emissions at different quantiles. For example, the ECI describes the positive association with environmental stress from lower to higher quantiles. Also, the outcomes regarding population aging & industrial development re-validate the prior outcomes across all quantiles. Furthermore, circular economy & carbon pollution describe the inverse connection with lower to upper quantiles emissions levels. Finally, PP negatively impacts environmental pollution but becomes insignificant at a higher quantile.

**Table 13:** Heterogeneity analysis by P-QR regression.

Variable	Q.15	Q.30	Q.45	Q.60	Q.75	Q.90
ECI	0.0392***	0.3485***	0.2239***	0.2392***	0.2332***	0.4733***
lnPA	7.4363***	12.304***	12.633***	10.993***	4.9194***	9.1254***
lnID	0.0624***	0.8689***	1.0311***	0.9717**	0.2033**	1.4060***
lnCE	−0.1223***	−0.1759***	−0.1882***	−0.2029***	−0.1256***	−0.0361***
lnCP	−1.0490***	−0.3217***	−0.7908***	−1.1005***	−0.6057**	−0.5303**
lnPP	−0.01301**	−0.1827***	−0.1930***	−0.17395**	−0.1693***	−0.0087*

Note: ***, **, and, * show the significance level at 1 %, 5 %, and 10, respectively.

## 5 5- Conclusion and policy recommendations

This empirical study introduces the critical determinants of environmental stress for 17 developed economies that have a leading role across the globe from 2000 to 2021. These factors include the economic complexity index, population aging, industrial development, public-private partnership, circular economy, and carbon policy. Similarly, to demonstrate the study's objective, this study uses the AMG, CCE-MG, CS-ARDL estimators, and panel Quantile regression for selected data. Summarized results describe the positive contribution of ECI, population aging, and industrial activities to environmental stress. However, circular economy, carbon policy, and PP significantly promote sustainability levels in specified countries. Due to the supportive response of key environmental solutions, the present study also offers a mediating effect on industrial development. Therefore, the mediating role of circular economy & carbon policy on industrial development significantly impacts emission reduction. Similarly, the mediating effect of public-private partnerships on industrial development shows an insignificant sustainability impact. Finally, this study offers some green insight to cover these flaws and protect the forthcoming generation.

### 5.1 Policy recommendations

From the policy perspective, this study executes several green suggestions to attain a sustainable environment shortly. Firstly, the critical role of ECI for selected economies is extraordinary, and shocking news compels policy analysts to propose green suggestions. The crucial importance of ECI has been for its economic and social benefits, while it has been neglected for its environmental sustainability. Therefore, it also has a pivotal role in environmental quality and the present research; it causes emissions in the long run. Thus, it is necessary to reshape development plans that deal with economic & social well-off and significantly support the green environment. Green industrial transition is highly recommended to deal with environmental harm. Similarly, specified economies must try to understand the channel where ECI may affect emissions levels, which may decline asymmetric information. Similarly, population aging also contributes to environmental damage in selected economies. There are a few suggestions to become clean & green shortly. Firstly, the aging population should focus on green growth that is favorable for long-term socio-economic progress. Similarly, the selected economies must focus on technology-intensive and low-emissions-intensive industrial activities to manage environmental harm. Similarly, specified economies should intervene at the domestic level to judge the key challenges that cause population aging to deteriorate environmental quality; in this way, these economies can resolve ecological issues. Moreover, it will be a rational choice if selected countries try to start a campaign in which they are rich in human knowledge to ensure equal priority for social, economic, and environmental aspects. However, green technologies and energies are highly recommended for becoming green shortly.

Furthermore, the industrial sector is also caused by environmental stress for the selected panel. In order to keep a clean & green environment, the chosen economies should take the following initiatives. For example, to attain sustainable development at minimum environmental cost, higher authorities must introduce environmental standards that the industrial sector must follow, such as efficiently utilizing available resources. Similarly, industrial development brings environmental pollution and causes biodiversity loss in its development procedure. Therefore, policy analysts must take care of industrial development plans to minimize carbon emissions. Similarly, due to the extensive cost of green energy utilization, the industrial sectors utilize traditional energy sources to meet production activities. It is necessary to offer green energy projects at a subsidized rate, which not only reduces the environmental cost but also boosts sustainable growth. Moreover, under the scenario of public-private partnership, countries try to invest in overall green projects because a single economic actor can simultaneously manage all problems. Additionally, there is a need to introduce a carbon policy for industrial activities that may help reduce emissions soon. The role of the circular economy in the industrial sector is also highly recommended for the selected panel. By revealing surprising behavior regarding circular economy (CE) and carbon emissions, the present study also suggests some green policies to improve the environmental quality further. Firstly, to overcome further environmental harm, the selected countries should check out the pick & drop process of garbage because careless transport activities in this process could bring massive biodiversity loss. Secondly, higher authorities must try to increase the knowledge of ordinary consumer to drop their wastage to the specified points instead of dropping at any space. Also, the technological development to convert energy from garbage is highly recommended and could help reduce over-dependence on fossil fuels and environmental sustainability. Moreover, environmental regulations via different tools have become a prominent method to deal with environmental stress. For instance, the green role of carbon policy in the selected economy also encourages policymakers to focus more on this regard. In recent years, the carbon tax has been utilized for two purposes: collecting tax for government revenue and green development projects. Therefore, carbon taxes should be allocated to green projects such as green energy projects rather than to utilize such revenue in government expenditures. Similarly, higher authorities must try to implement strict environmental laws along with carbon policy. Thus, due to solid regulations, the stakeholders may enhance the share of technical innovation and green energies in their production activities, which further causes sustainability. Also, the financial sector's support is highly needed to fight for environmental quality.

In addition, the current study also suggests some fundamental suggestions to strengthen the PP and its vital role in environmental sustainability. Firstly, there is a need to pay special attention to the innovation sector that could introduce innovative methods for industrial production activities, ultimately reducing environmental pressure. Similarly, the energy sector is also a leading factor that may cause environmental stress, but the public-private partnership in the energy sector could significantly improve sustainability levels via investment in green energy projects. Finally, energy-intensive industrial activities should be minimized, and policy analysts should promote PP participation in industrial activities to build a green environment.

### 5.2 Future limitations of the study

Similarly, the current study is not free from future limitations that future studies must address. Firstly, due to data constraints, the present study tries to investigate such an exciting theme only for the selected developed economies. Thus, it would be imperative if forthcoming studies try to utilize other vital factors, such as government stability, the rule of law, corruption, and external conflicts, which may influence sustainability. Moreover, due to specified objectives and data availability, this study considers only developed economies; if possible, future studies will consider other regions such as G7, E7, MENA, and Asian economies. Finally, this study utilizes a few estimators for empirical investigation; forthcoming studies can utilize other estimators to investigate such interesting themes and compare their outcomes with this study's outcomes.

## Funding

The authors declare that no funds, grants, or other support were received during the preparation of this manuscript.

## Ethics declarations

Not applicable.

## Ethical approval and consent to participate

Not applicable.

## Consent for publication

Not applicable.

## Data availability

Data will be available on special request.

## CRediT authorship contribution statement

**Daniel Balsalobre-Lorente:** Writing – original draft, Validation, Supervision, Conceptualization. **Syed Ale Raza Shah:** Software, Methodology, Formal analysis, Data curation. **Rena Huseynova:** Writing – review & editing, Validation, Resources.

## Declaration of competing interest

It is submitted that there are no known conflicts of interest associated with this publication that could have influenced its outcome.
